# The potential for vaccination-induced herd immunity against the SARS-CoV-2 B.1.1.7 variant

**DOI:** 10.2807/1560-7917.ES.2021.26.20.2100428

**Published:** 2021-05-20

**Authors:** David Hodgson, Stefan Flasche, Mark Jit, Adam J Kucharski, CMMID COVID-19 Working Group, Centre for Mathematical Modelling of Infectious Disease (CMMID) COVID-19 Working Group, Centre for Mathematical Modelling of Infectious Disease (CMMID) COVID-19 Working Group, Sam Abbott, W John Edmunds, Nicholas G. Davies, Rosalind M Eggo, Graham Medley, Jiayao Lei, Yang Liu, Damien C Tully, Ciara V McCarthy, Paul Mee, Akira Endo, Joel Hellewell, Sebastian Funk, Thibaut Jombart, Yalda Jafari, Oliver Brady, Kiesha Prem, Fabienne Krauer, Mihaly Koltai, Naomi R Waterlow, Timothy W Russell, Sophie R Meakin, Kathleen O'Reilly, Nikos I Bosse, William Waites, Emily S Nightingale, Rachel Lowe, Yung-Wai Desmond Chan, Katherine E. Atkins, Billy J Quilty, Frank G Sandmann, Kevin van Zandvoort, C Julian Villabona-Arenas, Hamish P Gibbs, James D Munday, Anna M Foss, Amy Gimma, Carl A B Pearson, Rosanna C Barnard, Matthew Quaife, Fiona Yueqian Sun, Alicia Rosello, Rachael Pung, Christopher I Jarvis, Emilie Finch, Kaja Abbas, Samuel Clifford, Gwenan M Knight, Simon R Procter

**Affiliations:** 1 Centre for Mathematical Modelling of Infectious Diseases, London School of Hygiene & Tropical Medicine, London, United Kingdom; 2 The members of the Working Group are listed below

**Keywords:** Herd Immunity, seroprevalence, SARS-CoV-2, vaccination

## Abstract

We assess the feasibility of reaching the herd immunity threshold against SARS-CoV-2 through vaccination, considering vaccine effectiveness (VE), transmissibility of the virus and the level of pre-existing immunity in populations, as well as their age structure. If highly transmissible variants of concern become dominant in areas with low levels of naturally-acquired immunity and/or in populations with large proportions of < 15 year-olds, control of infection without non-pharmaceutical interventions may only be possible with a VE ≥ 80%, and coverage extended to children.

Initial reports of vaccine effectiveness against severe acute respiratory syndrome coronavirus 2 (SARS-CoV-2), the virus responsible for coronavirus disease (COVID-19), have suggested a substantial reduction of the risk of infection [1]. Nevertheless, with the emergence of more transmissible variants such as B.1.1.7 [2], how large-scale immunisation programmes against SARS-CoV-2 will perform is currently unclear. This study assesses the potential of COVID-19 vaccination to generate herd immunity and takes into account vaccine effectiveness, naturally-acquired immunity and achievable vaccination coverage (depending on the population age structure), as well as two transmissibility scenarios ((i) with pre-B.1.1.7, and (ii) with exclusively B.1.1.7 variants).

## Vaccination and herd immunity

The feasibility of attaining vaccination-induced herd immunity depends on (i) vaccine effectiveness in reducing transmission, (ii) the transmissibility of the target pathogen and (iii) the vaccine coverage that is achievable in a population.

In a scenario where vaccines are distributed randomly across a population, the herd immunity threshold (HIT) for an immunisation programme is defined as 1 − 1/R_0_, where R_0_ is the basic reproduction number [3]. Note that if R_0_ is calculated using an age- or risk-structured next generation matrix then this equation will still hold. If vaccine effectiveness is below the HIT, then even vaccination of the entire population would, on its own, be insufficient to ensure control (i.e. the effective reproduction number, accounting for immunity, would remain above 1).

## Herd immunity, vaccine effectiveness and virus transmissibility

### Viruses with vaccines of varying effectiveness and different transmissibility

**Figure 1:**
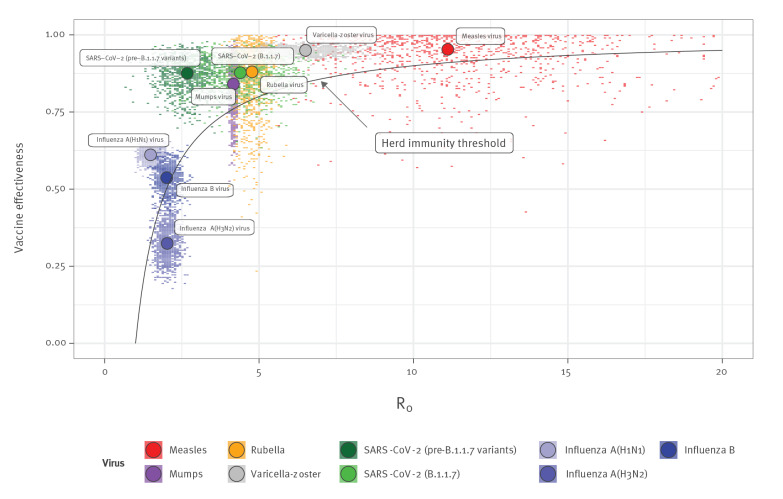
Comparison of effectiveness of currently available vaccines against herd immunity threshold for different viruses, 2000–2021 (n = 9 virus variants/types/subtypes)

Comparing this theoretical HIT with estimated values of R_0_ and vaccine effectiveness for a range of vaccine-preventable diseases (Figure 1), we see that for infections caused by viruses with little antigenic variation, vaccine effectiveness is sufficiently high to control transmission if high vaccine coverage is achieved. This is why, in many countries, childhood immunisation programmes have led to elimination of viruses with little antigenic variation and long-lasting sterilizing immunity, such as measles and rubella viruses [4].

SARS-CoV-2: severe acute respiratory syndrome coronavirus 2.

The black line shows the minimum vaccine effectiveness needed to achieve herd immunity for given R_0_ values. Colour points represent samples from available effectiveness and transmissibility estimates (see Supplement), with large circles showing medians. If sampled points are above the line, vaccination of the entire population could in theory lead to epidemic control; the more samples that are above the line, the higher the probability of control.

In contrast, viruses that undergo frequent antigenic turnover, such as influenza viruses necessitate regular vaccine updates and re-vaccination [5]. Seasonal influenza vaccine effectiveness is influenced by antigenic evolution of influenza viruses, with similar rates of evolution to that observed for seasonal human coronaviruses. Moreover, the effectiveness depends on whether the influenza vaccine strains are or not the same as the circulating ones. In the event of well-matched influenza vaccines, the effectiveness may nevertheless still be below the HIT. For influenza A(H3N2) virus, for example, the estimated effectiveness of an antigenically-matched vaccine (33%; 95% confidence interval (CI): 22–43) [6] implies that control of this subtype in the absence of natural immunity is unlikely, even in theory; we estimate a very small probability (defined as number of Monte Carlo samples) in an unexposed population with 100% vaccination coverage of being above the HIT.

### SARS-CoV-2

For SARS-CoV-2, we consider two SARS-CoV-2 variants for which we assume vaccination provides equal protection: pre-B.1.1.7 variants with an R_0_ of 2.7 (95% CI: 1.5–3.8) and the B.1.1.7 variant with an R_0_ of 4.5 (95% CI: 2.5–6.4) [7]. Assuming 86% (95% CI: 76–97) vaccine effectiveness against infectiousness, based on early estimates of protection against infection following two doses of Comirnaty (BNT162b2, BioNTech/Pfizer, Mainz, Germany/New York, United States) [1], we estimate, in the case of a pre-B.1.1.7 variant, a 99% probability of being above the HIT with whole-population coverage and a 94% probability if B.1.1.7 is circulating exclusively.

## Ethical statement

Ethical approval was not necessary for this modelling study as the analysis uses only aggregated secondary data from published articles.

## COVID-19 vaccination coverage achievable

The estimated 94% and 99% probabilities to be above the HIT, for B.1.1.7 and pre-B.1.1.7 SARS-CoV-2 respectively, are based on the assumption that the whole population is vaccinated. However, whole-population vaccination would require SARS-CoV-2 vaccines — which, as at mid-May 2021, are only approved for adults in most countries — to also be used at high coverage in children, for whom there is currently limited evidence from trials only, on safety or effectiveness.

Assuming a vaccination campaign aimed at all individuals ≥ 15 years old, the proportion of the population currently eligible for vaccination (i.e. comprising people aged ≥ 15 years) varies between countries, with the proportion of children within a country’s population decreasing along increasing income brackets (low, lower-middle, upper-middle, upper income). Given this trend, we use income level as a proxy for the total proportion of the population aged 15 years and over, which is eligible for vaccination.

## COVID-19 vaccination coverage needed considering natural immunity

Depending on vaccine coverage and effectiveness against infectiousness with future circulating variants – which may be antigenically dissimilar to B.1.1.7 [8] – herd immunity to SARS-CoV-2 in the absence of other non-pharmaceutical interventions may not be reached until considerable natural immunity has accumulated. As some countries now have a sizeable subpopulation with protective antibodies acquired through natural infection [9], we estimated the probability of reaching the HIT for SARS-CoV-2 under varying degrees of vaccination coverage against a background of reduction in transmission from natural immunity.

### Pre-B.1.1.7 SARS-CoV-2 transmission scenario

**Figure 2:**
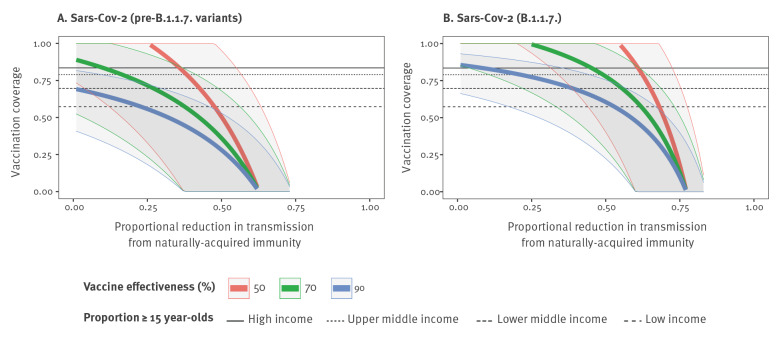
Vaccination coverage required to reach herd immunity for COVID-19, considering three different levels of vaccine effectiveness in (A) pre-B.1.1.7 and (B) B.1.1.7 SARS-CoV-2 transmission scenarios, 2021

COVID-19: coronavirus disease; CrI: credible intervals; SARS-CoV-2: severe acute respiratory syndrome coronavirus 2.

We estimated that for pre-B.1.1.7 SARS-CoV-2 variants, an immunisation programme targeting all persons aged ≥ 15 years (as would be the case for a vaccine not approved for use in younger groups), would have generated herd immunity against homotypic viruses in most higher income countries, regardless of the level of natural immunity, if vaccine effectiveness is at least 70% (or at least 90%, with a high degree of confidence). However, the high proportion of children in many lower income countries means the HIT cannot be reached with a ≥ 15-years-old vaccination programme alone and would require higher levels of immunity among children, acquired either through vaccination or infection, to be reached (Figure 2A).

Vaccination coverage required to reach herd immunity considering different levels of vaccine effectiveness and naturally-acquired immunity for SARS-CoV-2 in (A) a pre-B.1.1.7 transmission scenario and (B) a B.1.1.7 one.

Data sources are provided in the Supplement. Thick coloured lines show medians and shaded regions 95% CrI. Blue is the vaccine coverage necessary to reduce transmission when vaccine effectiveness is 90%; green, 70%; red, 50%. Black horizontal lines show the maximum vaccination coverage (i.e. when all people ≥ 15 years old are vaccinated) for different groups of countries, according to the proportions of ≥ 15 year-olds in their population, which are proxied by country income. The lower the country level of income, the lower the proportion of ≥ 15 year-olds in its population is, as well as the maximum vaccine coverage achievable. Maximum achievable vaccination coverage is averaged across all countries in particular income groups.

### SARS-CoV-2 B.1.1.7 transmissibility scenario

For B.1.1.7, or similarly transmissible variants, we would expect ongoing transmission until a sufficient level of natural immunity has been accrued, even in countries with an older age distribution. In our results, the estimated level of natural immunity to reach the HIT alongside vaccination varied considerably across countries, with high-income countries, which have a high proportion of adults, needing a 10% reduction in transmission from natural immunity on average, alongside widespread vaccination of all persons ≥ 15 years old, and low-income countries with a lower proportion of adults, needing a 53% reduction in transmission for the same vaccination programme (Figure 2B).

## SARS-CoV-2 seroprevalence as a proxy for natural immunity

We can use regional estimates of seroprevalence to approximate the proportional reduction in SARS-CoV-2 transmission due to natural immunity. For simplicity, we assume random mixing within a population, seropositivity as a perfect marker of immunity, and that natural immunity is fully sterilising (stops onward transmission). This is based on observations that seropositivity is associated with at least a 0.84 (95% CI: 0.81–0.87) reduction in risk of reinfection [10], and reinfections are likely to in turn be less infectious [11]. As seropositivity from natural infection is likely to be concentrated among groups more involved in transmission (i.e. seroprevalence would underestimate the corresponding reduction in transmission) this would also offset any contribution to transmission from individuals who have immunity that is not fully sterilising.

## Controlling SARS-CoV-2 spread in populations with different age structure and seroprevalence

**Figure 3:**
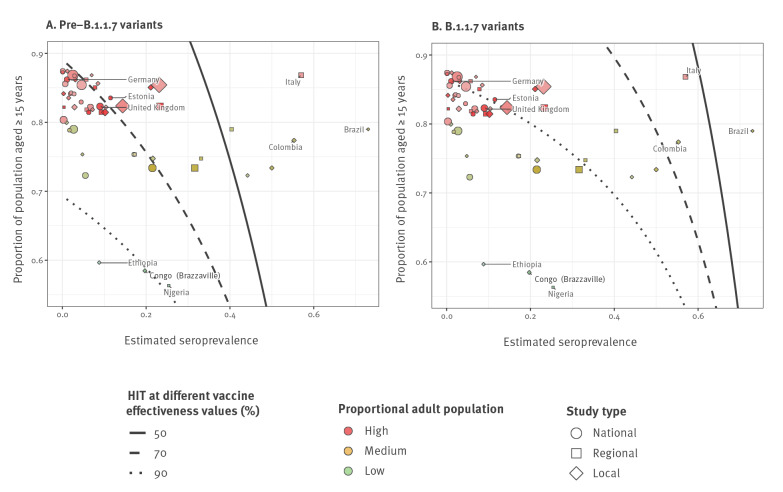
Estimated seroprevalence and eligible proportion for vaccination in different geographical areas, in relation to the herd immunity thresholds obtained with different vaccine effectiveness, considering (A) pre-B.1.1.7 and (B) B.1.1.7 SARS-CoV-2 transmission scenarios, seroprevalence estimates are from September 2020–April 2021

By assuming that the maximum vaccination coverage feasible is equal to the proportion of the population aged ≥ 15 years, we can therefore assess the feasibility in countries with varying proportions of adult population of reaching the HIT (Figure 3).

Adults: individuals aged ≥ 15 years; HIT: herd immunity threshold; SARS-CoV-2: severe acute respiratory syndrome coronavirus 2.

Panel (A) shows results considering pre-B.1.1.7 transmission while panel (B) shows results obtained with B.1.1.7 variant transmissibility.

By assuming that the estimated seroprevalence is equal to the proportional decrease in transmission because of natural immunity and that the proportion of adults is equal to the maximum achievable vaccination coverage, the plots show how feasible it is for various areas in the world to reach herd immunity against pre-B.1.1.7 or B.1.1.7 variants after a mass vaccination programme, for various vaccine effectiveness values.

The shape of the point marker represents the geographical scope of population used for the seroprevalence [12] estimate (national, regional or local) and the point marker radius scales with size of the study population.

The size of the point markers is proportional to the study size of the serosurvey.

The proportion of the population who are adults in the study site is given by colour hue: high category is above 80% (red), medium category is between 70 and 80% (orange), and low category is less than 70% (green) and the time since sample collection finished is shown through colour saturation, (i.e. high saturated points represent studies which were conducted more recently). The black line-types show the HIT thresholds for different vaccine coverage values. Seroprevalence data from serotracker.com.

We obtain seroprevalence estimates from SeroTracker [12], and population distribution estimates from The World Bank [13].

### Pre-B.1.1.7 SARS-CoV-2 transmissibility scenario

When pre-B.1.1.7 SARS-CoV-2 variants circulate, countries with a high proportion of persons ≥ 15 years old could theoretically reach the HIT through an adult mass immunisation programme with a vaccine effectiveness as low as 70%. For countries with a lower proportion of persons ≥ 15 years old, our results suggest adult immunisation campaigns alone would not be sufficient to provide vaccine-induced herd immunity if children contribute equally to transmission.

### SARS-CoV-2 B.1.1.7 transmissibility scenario

If the B.1.1.7 variant dominates, countries with a high proportion of adults require much higher effectiveness, ca 90%, to reach the HIT after an adult immunisation campaign, and many countries with a lower adult population proportion are again unlikely to reach the HIT through vaccination. Although there are some regional and local studies in countries such as Brazil [14], Italy [15], and Columbia [9] showing high seroprevalence at the local level, much lower seroprevalence has been estimated at the national level. For countries in which there is substantial heterogeneity in natural immunity, these estimates for herd immunity are unlikely to equate to a pragmatic protective threshold. However, estimates for herd immunity in such areas could be improved by developing models that include contact structure and geospatial clustering.

## Discussion and conclusion

This study considers the feasibility of reaching the herd immunity threshold against SARS-CoV-2 through vaccination and draws comparisons with other vaccine-preventable pathogens, including influenza and common immunising childhood infections.

Our observations suggest that if highly transmissible variants become dominant in areas with low seroprevalence, and/or in populations with a high proportion of children, control of infection by vaccination, in the absence of non-pharmaceutical interventions, may only be achievable with a vaccine effectiveness against infectiousness of ≥ 80% – as suggested by early data for the Comirnaty vaccine [1] – or next generation vaccines with persistently high effectiveness and cross-protection against antigenic variants, extended to the full population, including children.

As further vaccine effectiveness data emerge, our estimates of the potential for vaccination-induced-control could be further refined. Local differences in population age structure and behaviour, as well as biological characteristics of SARS-CoV-2 variants, could also change both baseline transmissibility between countries and which groups drive outbreaks [16]. If vaccine impact in reducing transmission is in reality higher than assumed here, the feasibility of local elimination would increase; for example, there is emerging evidence that infected vaccinees have a reduced risk of onward transmission (see Supplement). Conversely, future variants could reduce the effectiveness of current COVID-19 vaccines, and theoretical potential for local elimination, much as influenza vaccines are less effective against heterotypic strains [17]. In particular, while a highly transmissible homotypic SARS-CoV-2 variant would increase the herd immunity threshold, but to a value still below 100%, a sufficiently antigenically variable variant would mean herd immunity cannot be achieved with existing vaccines.

In the absence of booster campaigns and expanded coverage, herd immunity reached through a combination of vaccination and natural immunity could be short-lived. Therefore, our estimates are likely to be optimistic, as vaccine impact would decline as a result of increasing susceptibility from new births, waning protection and antigenic evolution (Supplemental Figure S1). However, some countries are considering third doses following their initial vaccination campaigns, which may counteract these effects [18].

As at mid-May 2021, vaccines are only approved for adults in most countries. If evidence on vaccine safety or effectiveness in children emerges from trials, the acceptability of vaccinating children, for whom an estimated 0.1 to 0.3% of symptomatic cases result in hospitalisation, may in practice be very different to adults, where between 1 and 30% of symptomatic cases result in hospitalisation, depending on age [19]. However, if such programmes are deemed feasible, overall vaccination impact could be increased if uptake is high among younger groups, such as young adults or older children, who contribute most to transmission [20].

In conclusion, based on current evidence, when considering reopening strategies, policymakers in countries with low seroprevalence or a high proportion of children should not assume that even vaccination of all adults will be sufficient to reach the herd immunity threshold. However, vaccination could still dramatically reduce the impact of SARS-CoV-2 infection on resulting disease within a population, particularly among groups at higher risk. This emphasises the importance, particularly in regions with limited vaccine-rollout capabilities and low seroprevalence, of public health measures and vaccination campaigns focused on reducing future COVID-19 disease burden, instead of relying on an assumption that transmission will necessarily be eliminated through vaccination.

## Code availability

The datasets generated during and analysed during the current study are available in the adamkucharski/hit-analysis repository, https://github.com/adamkucharski/hit-analysis.

## Supplementary Data

655000 Supplement
