# Supplementary material for: The potential for vaccination-induced herd immunity against the SARS-CoV-2 B.1.1.7 variant
Source: Euro Surveill. 2021 May 20;26(20):2100428. doi: 10.2807/1560-7917.ES.2021.26.20.2100428 (PMC8138959; doi:10.2807/1560-7917.ES.2021.26.20.2100428)
Supplement: Supplement [file 2100428_Supplement.pdf]

This supplementary material is hosted by Eurosurveillance as supporting information alongside the article *"The potential for vaccination-induced herd immunity against SARS-CoV-2 B.1.1.7 variant"* on behalf of the authors who remain responsible for the accuracy and appropriateness of the content. The same standards for ethics, copyright, attributions and permissions as for the article apply. Supplements are not edited by Eurosurveillance and the journal is not responsible for the maintenance of any links or email addresses provided therein.

Methods

Formula for required vaccine coverage

We defined the required coverage for herd immunity, C, as follows:

$$C = [(1 - 1/R_0) - p]/[(1-p) * V_e],$$

where R_0 is for SARS-CoV-2, p is the proportional reduction in transmission (due to previous infection), and V_e is the vaccine effectiveness. If R_0 is calculated using an age-structured next generation matrix and vaccination scales susceptibility across this matrix, the herd immunity threshold will still be $1–1/R_0$.

Source of vaccine effectiveness estimates

We obtained published estimates for the average and 95% upper and lower confidence intervals for vaccine effectiveness against measles[1], mumps[1], rubella[1], varicella[1], SARS-CoV-2[2], influenza A/H1N1 (post-2009), A/H3N2, and B[3]. For SARS-CoV-2, we used data from a study estimating vaccine effectiveness in reducing infection among antibody negative healthcare workers who received two doses of BNT162b2[4] (effectiveness was estimated at 86% (95% CI: 76-97%). This compares with an estimate of 83% (76-87%) lower risk of reinfection among healthcare workers following prior infection[5]. A recent analysis of UK community infection data[6] also estimated a 64% (95% CI: 55–70%) reduction in risk of infection following one dose of ChAdOx1 nCoV-19, and a 45% (33–54%) reduction in risk of transmission if infected after ChAdOx1 nCoV-19 vaccination[7], which would imply a potential reduction in transmission of around 70–85% post-vaccination, because P(transmit | exposed) = P(transmit | infected) x P(infected | exposed). In order to reflect uncertainty in estimates, we generated a set of Monte Carlo samples for each pathogen by fitting the average and upper/lower confidence intervals to a beta distribution and sampling 1,000 values.

Source of pathogen R_0 estimates

For mumps, rubella, and varicella, we obtained a set of 1,000 samples by bootstrapping a set of pre-vaccination R_0 estimates from various regions [8,9]. For measles, SARS-CoV-2 variants, and influenza subtypes, we obtained estimates of the average, upper confidence interval and lower confidence interval for each to a lognormal distribution in order to sample

1,000 values [10–13]. We assumed that transmissibility of SARS-CoV-2 B.1.1.7 was 67% higher than for pre-B.1.1.7 variants[11].

Source of age distribution data

Data on the country-specific proportion of the total population aged 0–14 years, and the income bracket of each country in 2020, was taken from The World Bank[14].

Source of sero-prevalence estimates

Seroprevalence studies were obtained from SeroTracker, a dashboard that synthesises findings from hundreds of global SARS-CoV-2 serological studies[15]. We aimed to estimate the seroprevalence in the general population for each study region. Therefore, in an attempt to reduce selection bias, we only considered prospective households/community studies. Each seroprevalence study provides information on study site country, sample size, geographical scope (national, region, local), and the time frame in which samples were collected. If multiple studies exist for a country within a geographical scope, we consider only the most recent estimate.

Supplementary Tables

Pathogen	Vaccine effectiveness (%, mean, 95% CI)	Basic reproduction number (mean, 95% CI)
Measles	96 (72–99)	12.0 (6.0–18.0)
Mumps	86 (65–92)	4.2 (3.6–4.5)
Rubella	89 (58–97)	4.7 (3.4–7.8)
Varicella	95 (92–97)	6.5 (3.3–16.9)
SARS-CoV-2 (pre-B.1.1.7)	86 (76–97)	2.7 (1.5–3.8)
SARS-CoV-2 (B.1.1.7)	86 (76–97)	4.5 (2.5–6.4)
Influenza A/H1N1 (post-2009)	61 (57–65)	1.4 (1.2–2.0)
Influenza A/H3N2	33 (22–43)	2.1 (1.6–2.5)
Influenza B	54 (46–61)	2.1 (1.6–2.5)

Supplementary Table 1: Assumed values of vaccine effectiveness and basic reproduction number for different pathogens, based on empirical estimates.

Supplementary Figures

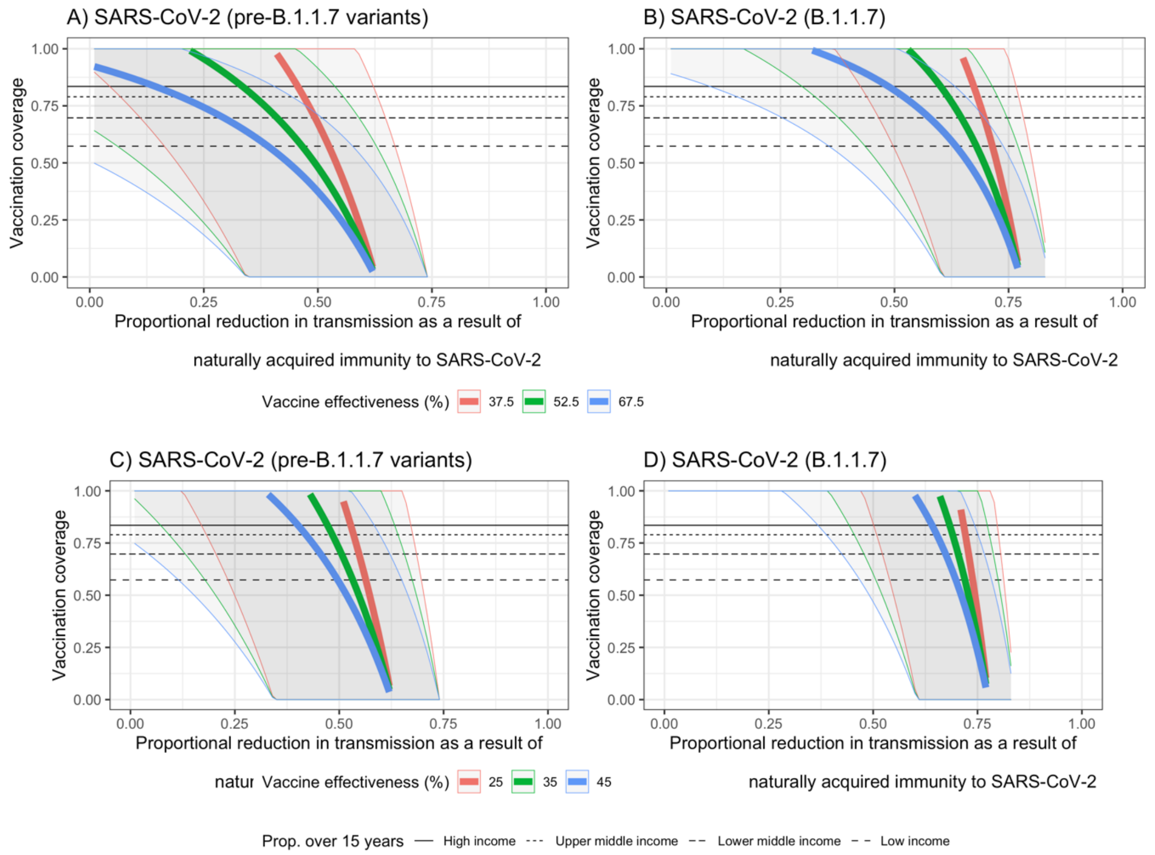

Figure S1. Vaccination coverage required to reach herd immunity for pre-B.1.1.7-like (A and C) and B.1.1.7-like (B and D) transmission and different levels of vaccine effectiveness assuming (in A and B) a reduction of 25% in vaccine effectiveness due to waning immunity, and (in C and D) a reduction of 50% of vaccine effectiveness due to warning immunity compared to vaccine effectiveness assumptions in **Figure 1b and 1c**.

References

1. Di Pietrantonj, C., Rivetti, A., Marchione, P., Debalini, M. G. & Demicheli, V. Vaccines for measles, mumps, rubella, and varicella in children. *Cochrane Database of Systematic Reviews* vol. 2020 (2020).
2. Weekes, M. *et al.* Single-dose BNT162b2 vaccine protects against asymptomatic SARS-CoV-2 infection. *Authorea Prepr.* (2021) doi:10.22541/AU.161420511.12987747/V1.
3. Belongia, E. A. *et al.* Variable influenza vaccine effectiveness by subtype: a systematic review and meta-analysis of test-negative design studies. *Lancet Infect. Dis.* **16**, 942–951 (2016).
4. Hall, V. J. *et al.* Effectiveness of BNT162b2 mRNA Vaccine Against Infection and COVID-19 Vaccine Coverage in Healthcare Workers in England, Multicentre Prospective Cohort Study (the SIREN Study). *SSRN Electron. J.* (2021) doi:10.2139/ssrn.3790399.
5. Hall, V. *et al.* Do antibody positive healthcare workers have lower SARS-CoV-2 infection rates than antibody negative healthcare workers? Large multi-centre prospective cohort study (the SIREN study), England: June to November 2020. *medRxiv* (2021) doi:10.1101/2021.01.13.21249642.
6. Pritchard, E. *et al.* Impact of vaccination on SARS-CoV-2 cases in the community: a population-based study using the UK's COVID-19 Infection Survey. *medRxiv* 2021.04.22.21255913 (2021) doi:10.1101/2021.04.22.21255913.
7. PHE. One dose of COVID-19 vaccine can cut household transmission by up to half. *Press Release* https://www.gov.uk/government/news/one-dose-of-covid-19-vaccine-can-cut-household-transmission-by-up-to-half (2021).
8. Edmunds, W. J., Gay, N. J., Kretzschmar, M., Pebody, R. G. & Wachmann, H. The pre-vaccination epidemiology of measles, mumps and rubella in Europe: Implications for modelling studies. *Epidemiol. Infect.* **125**, 635–650 (2000).
9. Nardone, A. *et al.* The comparative sero-epidemiology of varicella zoster virus in 11 countries in the European region. *Vaccine* **25**, 7866–7872 (2007).
10. Guerra, F. M. *et al.* The basic reproduction number (R0) of measles: a systematic review. *The Lancet Infectious Diseases* vol. 17 e420–e428 (2017).
11. Davies, N. G. *et al.* Estimated transmissibility and impact of SARS-CoV-2 lineage B.1.1.7 in England. *Science* (2021) doi:10.1126/science.abg3055.
12. Fraser, C. *et al.* Pandemic potential of a strain of influenza A (H1N1): Early findings. *Science (80-.).* **324**, 1557–1561 (2009).
13. Biggerstaff, M., Cauchemez, S., Reed, C., Gambhir, M. & Finelli, L. Estimates of the reproduction number for seasonal, pandemic, and zoonotic influenza: A systematic

review of the literature. *BMC Infect. Dis.* **14**, 480 (2014).
14. Population ages 0-14 (% of total population) | Data. https://data.worldbank.org/indicator/SP.POP.0014.TO.ZS.
15. Arora, R. K. *et al.* SeroTracker: a global SARS-CoV-2 seroprevalence dashboard. *The Lancet Infectious Diseases* vol. 21 e75 (2020).
